# Development of a Bayesian Network-Based Parallel Mechanism for Lower Limb Gait Rehabilitation

**DOI:** 10.3390/biomimetics10040230

**Published:** 2025-04-08

**Authors:** Huiguo Ma, Yuqi Bao, Chao Jia, Guoqiang Chen, Jingfu Lan, Mingxi Shi, He Li, Qihan Guo, Lei Guan, Shuang Li, Peng Zhang

**Affiliations:** 1 School of Information Engineering, Quanzhou Ocean Institute, Shishi City, Quanzhou 362700, China; zhwdrfcdaaerov@hotmail.com (H.M.); baoyuqi001@hotmail.com (Y.B.); jiachao119@hotmail.com (C.J.); lanjingfu001@hotmail.com (J.L.);; 2 School of Arts and Design, Yanshan University, Haigang District, Qinhuangdao 066000, China; cgq9691@ysu.edu.cn; 3 Department of Design, Kyungpook National University, Daegu 41566, Republic of Korea; smx0609@knu.ac.kr (M.S.); tayon13@sina.com (H.L.); 4 School of Art and Design, Wuhan University of Technology, Wuhan 430070, China; 349256@whut.edu.cn; 5 Datang International Power Generation Co., Ltd., Zhangjiakou Power Generation Branch, Zhangjiakou 075000, China; 15230605261@163.com

**Keywords:** lower limb rehabilitation training system, Bayesian network modeling, parallel mechanism control, kinematic analysis

## Abstract

This study aims to address the clinical needs of hemiplegic and stroke patients with lower limb motor impairments, including gait abnormalities, muscle weakness, and loss of motor coordination during rehabilitation. To achieve this, it proposes an innovative design method for a lower limb rehabilitation training system based on Bayesian networks and parallel mechanisms. A Bayesian network model is constructed based on expert knowledge and structural mechanics analysis, considering key factors such as rehabilitation scenarios, motion trajectory deviations, and rehabilitation goals. By utilizing the motion characteristics of parallel mechanisms, we designed a rehabilitation training device that supports multidimensional gait correction. A three-dimensional digital model is developed, and multi-posture ergonomic simulations are conducted. The study focuses on quantitatively assessing the kinematic characteristics of the hip, knee, and ankle joints while wearing the device, establishing a comprehensive evaluation system that includes range of motion (ROM), dynamic load, and optimization matching of motion trajectories. Kinematic analysis verifies that the structural design of the device is reasonable, aiding in improving patients’ gait, enhancing strength, and restoring flexibility. The Bayesian network model achieves personalized rehabilitation goal optimization through dynamic probability updates. The design of parallel mechanisms significantly expands the range of joint motion, such as enhancing hip sagittal plane mobility and reducing dynamic load, thereby validating the notable optimization effect of parallel mechanisms on gait rehabilitation.

## 1. Introduction

With advancements in rehabilitation technology and the increasing demand for personalized treatment, parallel multidimensional gait rehabilitation devices have become a key innovation in medical equipment [1]. As a fusion of modern rehabilitation medicine and advanced engineering, these devices transcend single-function limitations, influencing patients’ rehabilitation processes, motivation, and overall quality [2].

Traditional lower limb rehabilitation training device designs often emphasize the stability of the mechanical structure and the accuracy of the motion trajectory [3]. Traditional designs often neglect patients’ personalized rehabilitation needs and psychological experiences during the training process, as well as fail to fully consider the crucial role of advanced technologies, such as Bayesian networks, in optimizing rehabilitation training programs [4]. In fact, patients’ rehabilitation processes, training dynamics, and training effects are deeply influenced by the design of rehabilitation training devices. The design of parallel multidimensional gait lower limb rehabilitation training devices is actually a complex systems engineering project that spans multiple disciplines, including rehabilitation medicine, biomechanics, ergonomics, and Bayesian networks. It not only requires designers to understand patients’ rehabilitation needs and psychological states deeply but also to precisely grasp the scientificity and personalization of training programs, that is, by utilizing the principles of Bayesian networks through intelligent adjustment of training parameters and flexible switching of training modes, effectively guide patients to participate in training, optimize training effects, reduce physical and mental burdens, and thereby comprehensively enhance the level and depth of rehabilitation training [5].

Additionally, a significant approach to improving the design of rehabilitation devices lies in the integration of biomimicry principles. By emulating the natural motion and coordination of muscles, joints, and tendons, rehabilitation devices can better replicate human gait patterns. This biomimetic approach enhances device comfort and functionality, supporting the body’s healing processes by mimicking real-life movements. Ultimately, such designs optimize rehabilitation outcomes by making the process more efficient and intuitive.

To address the complexity of personalized rehabilitation needs and training variability, this study proposes a dynamic Bayesian network model. It analyzes probabilistic dependencies among rehabilitation scenarios, joint motion deviations, and goals, dynamically optimizing training parameters for personalized rehabilitation. This approach establishes a systematic design framework that enhances training efficiency and personalization. Integrating Bayesian networks with advanced design methods enables intelligent adaptation to patient needs, improving rehabilitation outcomes. Ultimately, this strategy advances multidimensional gait rehabilitation devices, driving rehabilitation equipment toward greater personalization, intelligence, and effectiveness.

## 2. Related Research

Bayesian networks, as a powerful probabilistic graphical model, are an effective tool for addressing problems of uncertainty and complexity. They combine the strengths of graph theory and probability theory, using directed acyclic graphs (DAGs) to intuitively represent dependencies between variables and using conditional probability tables (CPTs) to quantify these relationships. In Bayesian networks, nodes represent random variables, edges indicate direct dependencies between variables, and CPTs describe the probabilities of a child node entering various states given the states of its parent nodes [6].

Over the past decade, Bayesian network models have emerged as key tools for analyzing complex systems, thanks to their probabilistic reasoning. They have simplified knowledge management in fields dealing with uncertainty and probabilistic data [7]. For example, Bayesian networks offer a knowledge representation scheme that describes design objects based on probabilistic dependencies among various system components. Similarly, variant models based on Bayesian networks have been used to describe and predict the behavior and states of design objects through probabilistic reasoning [8]. Chao Yu et al. [9] noted that reinforcement learning demands numerous trial-and-error interactions, while Bayesian networks personalize parameter adjustments via probabilistic inference, lessening patients’ physical strain. Tao Shen et al. [10] showed that Bayesian networks excel over traditional regression models in handling multi-joint interaction uncertainties in gait rehabilitation. Recently, several similar models and methods have been proposed to further enrich the application of Bayesian networks in the design of rehabilitation training devices [11]. For instance, to assist creative design activities during the conceptual design phase, a design method combining dynamic Bayesian networks with a human fatigue assessment scheme has been proposed, supporting design decisions through probabilistic reasoning [12]. To more effectively capture and manage design knowledge, a modeling method comprising instance models, functional models, and dynamic models has been introduced, with Bayesian networks serving as the core tool for knowledge representation and reasoning [13].

In the field of rehabilitation training device design, Bayesian networks and their variants are also widely used to describe and reason about the characteristics of design objects. Specifically, some studies have applied Bayesian networks to the functional modeling of rehabilitation training devices, revealing the intrinsic logic of design objects by representing probabilistic relationships between functions [14]. At the same time, Bayesian networks are also used to support analogical design, aiding decision-making by comparing probabilistic distributions among different design schemes [15]. Additionally, scholar Guo Jian has conducted in-depth research on Bayesian networks in multiple aspects. They have explored the advantages of Bayesian networks in handling uncertainty in complex systems, proposed more efficient reasoning algorithms, and provided a theoretical foundation for the application of Bayesian networks in the design of rehabilitation training devices [16]. Scholars such as Li Gang have combined Bayesian networks with machine learning techniques, optimizing network structures through training data to improve the accuracy and generalization ability of the models [17]. Based on these basic models, further research has addressed more specific problems by adding more dimensions. For example, a contextual Bayesian network framework has been proposed, attempting to extend traditional Bayesian network models to consider the dynamic characteristics and contextual information of the design environment [18]. Furthermore, although Bayesian networks initially focused on representing static probabilistic relationships, a Bayesian network ontology for representing dynamic processes has been developed, providing a unified framework for process classification and incorporating higher-level semantics in its representation [19]. However, the application of existing dynamic Bayesian networks in the field of rehabilitation medicine still has significant limitations. Their dynamic modeling mostly focuses on state transitions within a single time slice, failing to fully integrate the temporal correlations and cross-dimensional interaction characteristics of multi-joint motion data in rehabilitation training [20]. Additionally, existing frameworks are inadequate in adapting to patients’ personalized rehabilitation needs, such as the lack of a dynamic adjustment mechanism for rehabilitation priorities and functional impact weights, making it difficult for training programs to precisely match the goals of different pathological stages [21]. In complex rehabilitation scenarios (such as multidimensional gait compensation), traditional models cannot synergistically optimize mechanical system kinematics constraints and human biomechanical characteristics, limiting their practical application effects [22].

**Figure 1:**
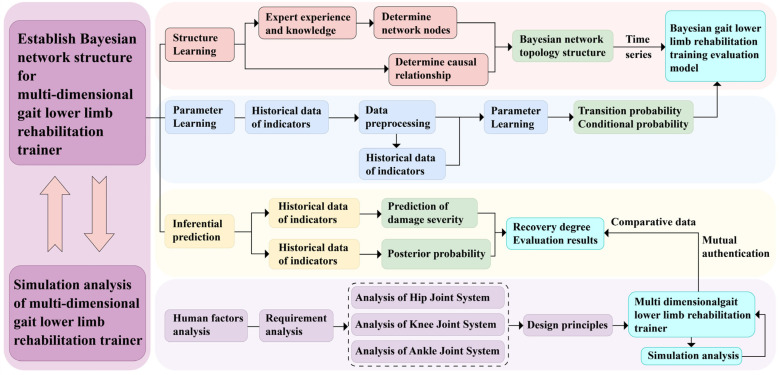
Research framework diagram of a Bayesian network for a multidimensional gait lower limb rehabilitation training device with a parallel structure.

By reviewing previous research and its development, it can be observed that the primary focus has always been on how to use Bayesian networks to capture and represent probabilistic dependencies between design objects. To achieve specific goals, scholars such as Siyi have added several additional elements based on these basic elements [23]. The design model for a parallel-structure multidimensional gait lower limb rehabilitation training device based on Bayesian networks proposed in this study is shown in Figure 1, adopting some methods introduced in previous research, with Bayesian networks serving as the core for describing the basic components of the design object. Compared with previous research, this study innovatively combines Bayesian networks with mechanical structures, not only considering human uniqueness from an ergonomic perspective more deeply but also fully leveraging the probabilistic reasoning capabilities of Bayesian networks to provide personalized rehabilitation service plans for patients, which is a significant innovation of this thesis. In traditional rehabilitation devices, static models often fail to account for the dynamic nature of patient progress and the variability in rehabilitation outcomes. This study’s integration of Bayesian networks addresses this limitation by continuously updating the probabilistic relationships between patient data and rehabilitation goals. This dynamic adjustment allows the rehabilitation process to adapt in real time, offering personalized and evolving rehabilitation plans based on the patient’s changing condition. As user-oriented design becomes more common, needs analysis is increasingly important. It also involves design evolution, referring to changes and improvements throughout the design process. More importantly, it explains how Bayesian networks integrate design knowledge during this evolution, enabling effective knowledge capture and reuse. Additionally, it considers how to combine tacit and formal knowledge to enhance design efficiency and effectiveness.

## 3. Patient-Centric Bayesian Network Modeling

### 3.1. Construction of Bayesian Network Structural Model

To construct a Bayesian network model for the design of a multidimensional gait lower limb rehabilitation training device, the core lies in accurately determining the network’s topology and the conditional probabilities of each network node. The structure of the dynamic Bayesian network (DBN) is organized by identifying the factor nodes that influence the rehabilitation process and the causal relationships between them. In practical rehabilitation applications, this involves mapping out key factors such as joint movements and recovery stages and using expert knowledge to determine how they interact to optimize training outcomes.

Target node variable set = {rehabilitation training scenario (E) → mild repair, moderate repair, severe repair}. This target node represents different levels or stages of rehabilitation training and is a core indicator for assessing the effectiveness of rehabilitation training.

Intermediate node variable set = {hip sagittal plane (B1), hip coronal plane (B2), hip horizontal plane (B3), knee sagittal plane (B4), knee horizontal plane (B5), ankle sagittal plane (B6), ankle coronal plane (B7), ankle horizontal plane (B8) → recovery condition, injury condition}. For instance, in a practical rehabilitation case, if a patient sustains a knee injury, monitoring the movement in the knee sagittal plane (B4) during gait training is crucial. Restricted or abnormal movement may signify poor recovery or an unresolved injury. These intermediate nodes represent the movement states of various lower limb joints in different planes, as well as their recovery or injury status during the rehabilitation process, which are key factors influencing the effectiveness of rehabilitation training.

Observation node variable set = {flexion (hip) (B11), extension (hip) (B12), abduction (hip) (B21), adduction (hip) (B22), external rotation (hip) (B31), internal rotation (hip) (B32), flexion (knee) (B41), external rotation (knee) (B51), internal rotation (knee) (B52), flexion (ankle) (B61), extension (ankle) (B62), abduction (ankle) (B71), adduction (ankle) (B72), external rotation (ankle) (B81), Internal rotation (ankle) (B82)}, and rehabilitation goals (P1, P2) = {rehabilitation goal (P1) → gait improvement, strength enhancement, flexibility recovery; rehabilitation goal (P2) → coordination enhancement, strength increase, flexibility improvement; rehabilitation goal (ankle) (P3) → balance improvement, motor ability enhancement, physical fitness improvement}. These observation nodes represent specific joint movements and rehabilitation goals, which are data that can be obtained through actual measurements or assessments and are used to infer the states of intermediate nodes and target nodes.

**Figure 2:**
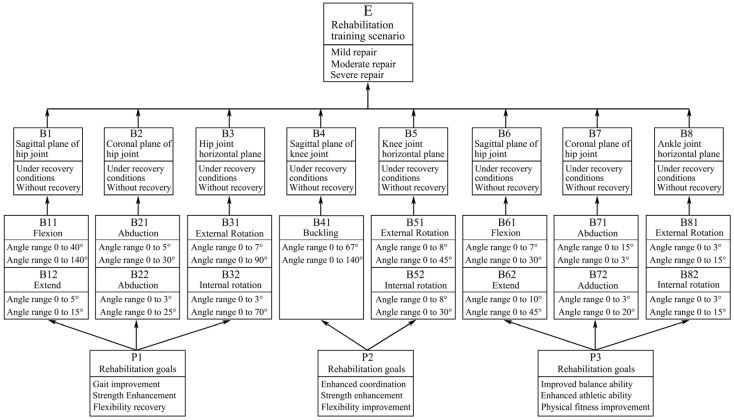
Bayesian network structure of the multidimensional gait lower limb rehabilitation trainer with a parallel structure.

After determining the DBN node variables, the time slice interval is set to a single training cycle (20 min), and the node state transition probabilities are updated using a sliding window algorithm to reflect the dynamic dependencies in the patient’s rehabilitation progress in real time. The observation nodes are considered the parent nodes of the network, the intermediate nodes serve as bridges connecting the observation nodes and the target nodes, and the target nodes are the child nodes of the network. Next, using professional Bayesian network construction tools such as GENIE or other similar software, the dynamic Bayesian network topology for the design of the multidimensional gait lower limb rehabilitation training device can be quickly constructed, as shown in Figure 2.

Through this structure, we can intuitively observe the interaction relationships among various influencing factors and how they jointly affect the rehabilitation effect of the trainer. For example, different joint movements (observation nodes) can influence the joint’s motion states in different planes (intermediate nodes), thereby determining the rehabilitation training scenario (target node). At the same time, rehabilitation goals (P1, P2, P3 in the observation nodes) also have a significant impact on the process and effectiveness of rehabilitation training.

After analysis and processing by the software, a Bayesian network evaluation model for the design of the multidimensional gait lower limb rehabilitation trainer was ultimately obtained. This model not only demonstrates the complex relationships among various influencing factors but also provides us with a basis for quantitatively evaluating the rehabilitation effect of the trainer, offering strong support for subsequent optimization design and the formulation of personalized rehabilitation programs.

### 3.2. Bayesian Network Computation and Analysis

**Figure 3:**
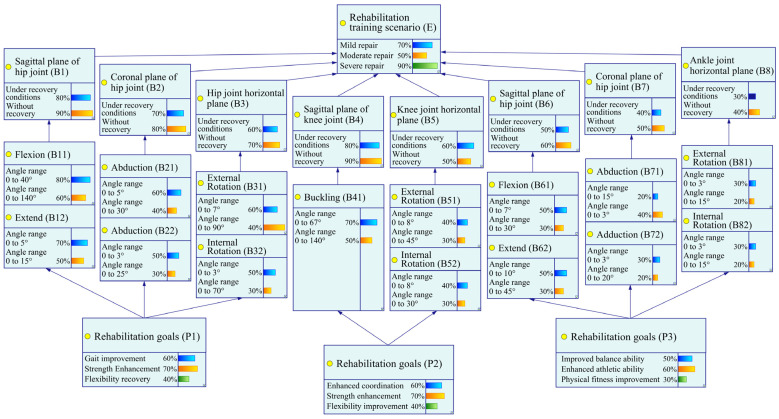
Node status.

To comprehensively obtain relevant data, this study conducted a 6-week survey on 200 rehabilitation patients with lower limb movement disorders, including hemiplegia and stroke. The survey employed a combination of multiple methods, including observation, questionnaire surveys, and professional gait analysis techniques. In terms of observation, we recorded detailed training behavior data of the patients, including training conditions, postures, durations, rest periods, and training responses, within the limit of their weekly training hours. We also used cameras and motion capture systems to film the entire process, ensuring the accuracy and completeness of the data. After the observation, the data were statistically organized to construct a behavior database for rehabilitation trainers in Figure 3. Regarding the questionnaire surveys, we designed a questionnaire scale based on the observed data, covering dimensions such as force application experience, training difficulty, duration, rest, equipment comfort, training effect, etc. Patients were asked to provide subjective descriptions and ratings to understand further their experiences and perceptions of using the rehabilitation trainer. Additionally, considering the specificity of the training conditions, this study employed professional gait analysis techniques to conduct an in-depth analysis of the patients’ training postures and gaits. Gait analysis techniques provided detailed data on patients’ gait parameters, muscle activity, joint angles, etc., serving as an important basis for subsequent Bayesian network modeling. During the data preprocessing stage, due to the mathematical principles of Bayesian networks requiring network nodes to be composed of discrete variables for improved accuracy, this study discretized all collected data. Specifically, all nodes were processed as binomial nodes with discrete data values {0, 1}. Here, 0 represents a “good” state, and 1 represents a “poor” state. This discretization process ensured data consistency and comparability, laying a solid foundation for subsequent Bayesian network modeling and analysis.

By introducing the two core concepts of functional impact weight and rehabilitation priority, this study not only clearly defined the key functional nodes in the rehabilitation process and the rehabilitation goals that should be achieved at different stages but also provided effective means for quantitatively assessing rehabilitation effects. This provided a scientific basis for making rehabilitation decisions, optimized the allocation of rehabilitation resources, stimulated patients’ active participation, and comprehensively improved the scientificity and effectiveness of rehabilitation treatment. Functional impact weight (weight range: 1–5) is an important indicator for assessing the contribution of each rehabilitation node to the overall rehabilitation goal. Core functional nodes, such as hip sagittal plane motion (B1) and knee sagittal plane motion (B4), play a crucial role in achieving the rehabilitation goal and are therefore assigned the highest weight of 5. Secondary functional nodes, such as ankle coronal plane motion (B7), contribute to the rehabilitation goal to some extent but are of slightly lower importance and are assigned a weight of 3. Auxiliary functional nodes, such as hip abduction motion (B21), although having relatively small direct contributions to the rehabilitation goal, cannot be ignored in the overall rehabilitation process and are therefore assigned the lowest weight of 1.

Rehabilitation priority (priority coefficient range: 0.1–1.0) dynamically adjusts the priority order of various rehabilitation items or goals based on different rehabilitation stages. In the mild recovery stage, the main goal is to restore the joint range of motion, such as flexion (B11) and extension (B12) movements, which are assigned a higher priority coefficient of 0.8. Entering the moderate recovery stage, the main goals shift to enhancing strength and improving coordination, such as rehabilitation goals P1/P2, at which point the priority coefficient is adjusted to 0.6. In the severe recovery stage, the main goals are to improve flexibility and enhance balance abilities, such as ankle horizontal plane motion (B8), which is assigned a lower priority coefficient of 0.4.

The prior probability distribution of the hip joint observation node is shown in Table 1.

Based on the prior probability distribution data provided in Table 1, Table 2 and Table 3, we can observe the probability distribution of each joint under different states. Taking the hip joint as an example (Table 1), the probability distribution of states 0 and 1 for angle ranges such as flexion, extension, abduction, adduction, external rotation, and internal rotation shows the probability of the joint state in patients before rehabilitation training. These data provide a basic reference for the design of the training device, helping to understand the limited joint function of patients in their initial state. Similarly, the prior probability distributions of the knee joint (Table 2) and ankle joint (Table 3) also show the probability distribution of patients under different rehabilitation goals such as improved coordination, increased strength, and improved flexibility. These data reflect the joint function status of patients before the start of rehabilitation training and provide a basis for the formulation of training plans. Table 4 presents the posterior probability distribution of rehabilitation goals for the hip, knee, and ankle joints under different rehabilitation training scenarios (mild, moderate, and severe repair). By comparing the prior and posterior probabilities, we can observe the impact of rehabilitation training on the recovery of patients’ joint function.

In the mild repair scenario for the hip joint, the probability of gait improvement is 0.410, and the probabilities of strength enhancement and flexibility recovery also change, reaching 0.350 and 0.240, respectively. As the intensity of rehabilitation training increases (moderate and severe repair), although the probabilities of each goal fluctuate, the overall trend shows a gradual recovery of patients’ joint function. The probability of improved coordination for the knee joint is 0.416 in the mild repair scenario, and this probability remains relatively stable as the training intensity increases, indicating that the training device has a certain effect in improving knee joint coordination. The probabilities of strength enhancement and flexibility improvement also show a positive trend. The probability of improved balance ability for the ankle joint is 0.278 in the mild repair scenario, and this probability gradually increases to 0.312 (severe repair) as the training intensity increases, indicating that the training device has a significant effect on improving ankle joint balance ability. The probabilities of enhanced motor ability and physical fitness improvement also show a positive correlation.

By comparing the prior and posterior probability distributions, it is evident that rehabilitation training significantly improves the probability of functional recovery in patients’ hip, knee, and ankle joints, especially in terms of gait improvement, strength enhancement, flexibility recovery, coordination enhancement, and balance ability improvement. In terms of training intensity and effect, as the intensity of rehabilitation training increases, the probability of patients’ joint function recovery shows a positive correlation, indicating that the design of the training device can effectively promote the recovery of patients’ lower limb function. In terms of the effectiveness of the training device, the design of the parallel structure multidimensional gait lower limb rehabilitation training device based on a Bayesian network has shown significant effects in promoting the recovery of patients’ lower limb function, verifying the effectiveness and practicality of the training device. Through data analysis of the Bayesian network model, the effectiveness of the design of the parallel structure multidimensional gait lower limb rehabilitation training device based on a Bayesian network has been verified. The training device has shown significant effects in promoting the recovery of patients’ lower limb function, especially in terms of improving joint function, enhancing coordination, and improving balance ability.

## 4. Design and Evaluation of a Multidimensional Gait Lower Limb Rehabilitation Trainer

### 4.1. Design Considerations for Reducing Fatigue in Gait Rehabilitation Devices

#### 4.1.1. Analyzing Design Requirements for Fatigue Prevention

The design of the multidimensional gait lower limb rehabilitation trainer, based on a parallel structure utilizing Bayesian networks, must fully consider the need for fatigue prevention during the rehabilitation training process while also focusing on the device’s functionality and efficiency. Compared with traditional lower limb rehabilitation training equipment, the design of this study needs to balance the protective effectiveness of the trainers and the comfort of wearing and using the device. Therefore, we have adopted a “user-centered design (UCD)” approach in the design process [24], as a substitute for the traditional mechanical equipment design mindset.

“User-centered design” is the core idea of human ergonomics research, emphasizing the placement of human needs and capabilities at the center of the design technology system to achieve overall optimization of the “human–machine–environment” system, thereby attaining the goals of “health, safety, comfort, and efficiency.” The International Organization for Standardization released the basic process for “user-centered design” [25] as early as 1999 and made the latest revision in 2019 [26]. This process can effectively introduce ergonomic methods into the design process, improve design efficiency and effectiveness, and ensure human health, safety, and performance. This process can serve as a guiding principle for the fatigue prevention design of the wearable lower limb rehabilitation trainer in this study [27].

Gould et al. summarized the three core principles of “user-centered design” [28]:

(1) Mandatory focus on users and tasks;

(2) Quantitative testing;

(3) Iterative design.

Correspondingly, Maguire further divided “user-centered design” into five stages [29]:

(1) Formulating a user-centered design plan;

(2) Clarifying the usage context;

(3) Specifying user needs;

(4) Product design;

(5) Design evaluation.

The rational application of the “user-centered design” approach can effectively enhance the usability and user satisfaction of the multidimensional gait lower limb rehabilitation trainer [30]. In this study, “usability” encompasses two meanings: first, trainers can use the device without specialized learning; second, trainers can independently complete the wearing and adjustment of the device.

Based on the above discussion, when analyzing the design requirements for human fatigue prevention in the multidimensional gait lower limb rehabilitation trainer, this study first selected a certain number of patients with lower limb movement disorders and rehabilitation medical experts and provided detailed descriptions of their fatigue prevention needs during the rehabilitation training process. Subsequently, the importance of these needs was rated using a Likert five-point scale, with the specific statistical results shown in Table 5.

As can be seen from Table 5, needs such as lower limb support, reduction of joint burden, and alleviation of lumbar and back muscle fatigue have been assigned high priority. In addition, factors such as comfort, adjustability, breathability, and ease of operation of the device are also key considerations in the design process.

On the other hand, by combining the reasoning results of rehabilitation training data using a Bayesian network, we can observe differences in patient fatigue levels under different training modes. For example, in high-intensity or long-duration training modes, patients’ lower limb and lumbar muscles are more prone to fatigue. Therefore, during the fatigue prevention design process, enhanced protection for the relevant muscle groups should be implemented for these specific training modes to ensure patient safety and comfort during rehabilitation training. This aligns with the results of the aforementioned design needs analysis and provides an important reference for subsequent design work.

#### 4.1.2. Overall Analysis of the Lower Limbs

The lower limbs, as the core focus of rehabilitation training, have structural and functional characteristics that are crucial for the design of the trainer. From an anatomical perspective, the lower limbs primarily consist of the trunk (here simplified as the part connected to the hip joint), the thigh, the calf, and the foot, which are sequentially connected through the hip joint, knee joint, and ankle joint, forming a complex and flexible movement system.

From a mechanical design standpoint, we can liken the various parts of the lower limbs to linkages in robotics, with the hip joint, knee joint, and ankle joint playing the roles of kinematic pairs. To simplify the analysis, we can approximate the hip joint and ankle joint as spherical joints capable of rotating around three mutually perpendicular axes in space. Although in actual movement, these joints do not purely rotate but also involve a certain amount of translational motion, in most cases, this translational component is relatively small. It can be neglected, thereby simplifying these two joints into spherical hinge motions.

The situation with the knee joint is slightly more complex. Due to the limited range of abduction–adduction motion in the coronal plane and internal–external rotation motion in the horizontal plane, we can simplify its motion into a single-degree-of-freedom rotational motion with a changing axis primarily within the sagittal plane. This simplification helps us to more clearly understand the movement patterns of the knee joint during gait and provides a theoretical basis for the design of the rehabilitation trainer.

The range of motion of each joint is protected and limited by connective tissue and ligaments, which sets certain limits on the rotation amplitude of each joint. At the same time, there are differences in the rotation amplitude of the same joint along different directional axes and in the rotation amplitude among different joints. Based on biomedical observations and statistics, we can obtain the extreme range of motion angles and normal gait motion angle ranges for each joint of the human lower limbs [31], with the results shown in Table 6.

An analysis of these data reveals that the range of motion varies among different joints in the human body, and normal gait movement primarily occurs within the sagittal plane, with relatively less movement in the coronal and horizontal planes. There is a significant difference between the range of gait movement and the physiological range of motion, indicating that relying solely on normal gait movement and simple movements within the sagittal plane for rehabilitation training has certain limitations and may not adequately stimulate all joints. Therefore, when designing a multidimensional gait lower limb rehabilitation training device with a parallel structure, it is necessary to fully consider the motion characteristics and range of motion of each joint in the lower limbs to ensure that the device can provide comprehensive and effective rehabilitation training.

### 4.2. Equipment Design of the Multidimensional Gait Lower Limb Rehabilitation Training Device

#### Design of the Lumbar and Back Rehabilitation Structure

**Figure 4:**
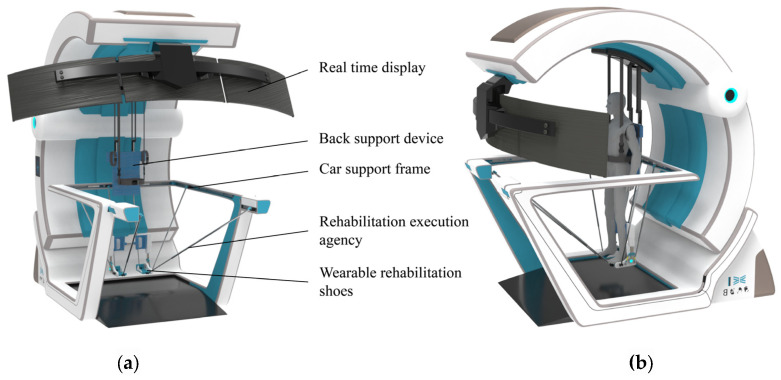
Mechanical structure model. (**a**) Structural composition. (**b**) Wearing effect.

The lower limb rehabilitation robot consists of four parts: a back support device, a trolley support frame, a rehabilitation actuator, and wearable rehabilitation shoes. The mechanical model of the device is shown in Figure 4.

Considering the motor impairments in patients’ lower limbs and their difficulty in maintaining balance during rehabilitation, an auxiliary rehabilitation trolley is designed to provide stable support for patients. The trolley is equipped with a back support device that features straps similar to a backpack for easy wearing, allowing the upper limbs to be relatively fixed to facilitate assisted support. The back support device also has a lifting function, enabling patients to transition from a sitting to a standing position for standing rehabilitation. The trolley is equipped with lockable casters for easy movement of the entire device. Patients can perform gait rehabilitation with the wheels unlocked or switch to other rehabilitation modes with the wheels locked, enhancing rehabilitation convenience without occupying fixed space.

**Figure 5:**
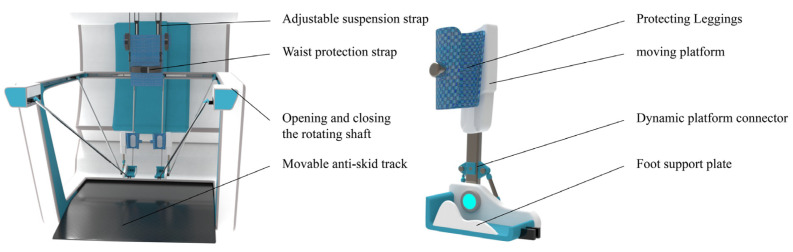
Mechanical structure model.

The human ankle joint can be approximately regarded as a spherical joint. Due to the natural range of motion of the human joints, constraining the ankle joint during rehabilitation and relying solely on three translational degrees of freedom may cause discomfort. Therefore, two local degrees of freedom are added to the ankle constraint design to accommodate the ankle’s motion in the sagittal and coronal planes. To improve the overall comfort of the rehabilitation, two planes are used at the bottom of the foot to ensure support, matching the bending degree of freedom of the foot during gait rehabilitation. The partial structure of the shoe is shown in Figure 5.

By securing the adjustable straps to the calf, the moving platform connector and the moving platform form a revolute joint perpendicular to the coronal plane to accommodate ankle abduction and adduction. The foot support plate and the moving platform connector form a revolute joint perpendicular to the sagittal plane to achieve ankle flexion and extension.

## 5. Kinematic Analysis of the Rehabilitation Actuator

### Inverse Kinematics of the Rehabilitation Mechanism

**Figure 6:**
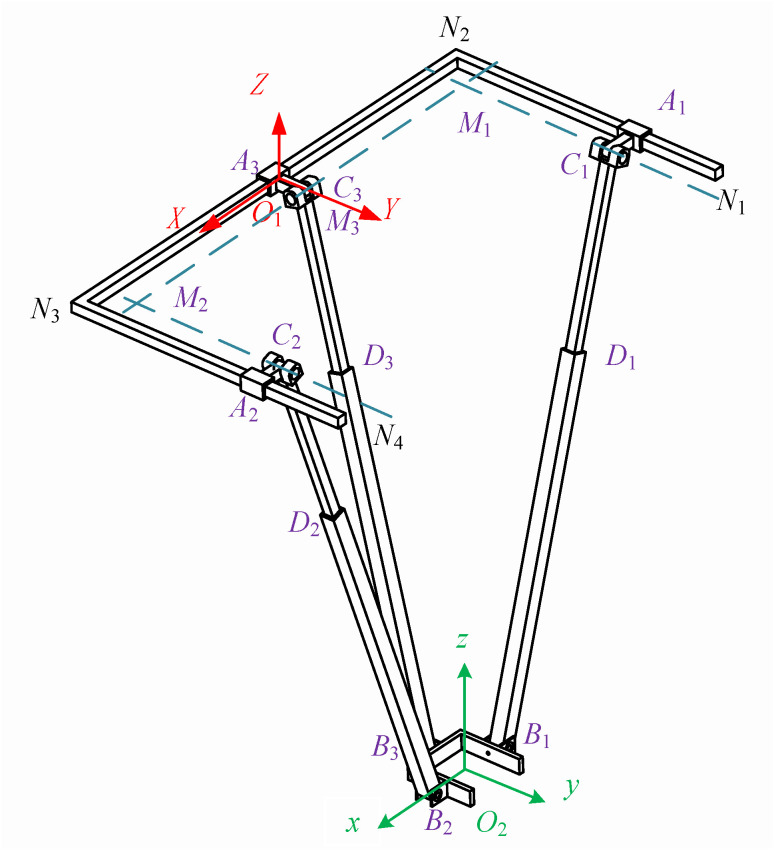
Diagram of the rehabilitation actuator.

Kinematic analysis is a crucial theoretical foundation for workspace analysis and trajectory planning. Therefore, a kinematic model is established for the rehabilitation actuator to analyze its kinematic characteristics. Since the same rehabilitation actuator is used for both the left and right legs, kinematic analysis is only required for a single-side rehabilitation mechanism. A diagram of the single-side rehabilitation actuator is shown in Figure 6.

Upon analyzing the mechanism, it is found that the number of components *n* = 11, the number of kinematic pairs g = 12, and the total number of kinematic pairs sum up to $\sum_{i=1}^{g}f_{i}=12$. The mechanism has over-constraints μ = 3. Therefore, according to the degree of freedom calculation, Formula (1) is as follows:

(1) $$M=6(n-g-1)+\sum_{i=1}^{g}f_{i}+\mu$$

In the formula:

*M*—degrees of freedom of the mechanism

*n*—number of components

g—number of kinematic pairs

$f_{i}$—degrees of freedom of the i-th kinematic pair

*μ*—number of redundant constraints

$M=6(n-g-1)+\sum_{i=1}^{g}f_{i}+\mu$ The degree of freedom of the mechanism is obtained as follows: M=6(11−12−1)+12+3=3; B1, B2, and B3 are selected as the moving platform of the mechanism, which has three-dimensional translational degrees of freedom in space.

In Figure 6, the moving platform B1, B2, and B3 serves as the output platform of the mechanism. It is connected to the fixed platform N1, N2, N3, and N4 through three PRPR branches, namely A1, C1, D1, and B1; A2, C2, D2, and B2; and A3, C3, D3, and B3. Both the moving and fixed platforms can be visualized as having a “匚”-shaped structure. The moving platforms B1, B2, and B3 are attached to the human ankle joint.

Before solving for the inverse kinematics, it is necessary to establish a coordinate system. Firstly, a base coordinate system fixed to the fixed platform is established $O_{1}-XYZ$; a coordinate system $O_{2}-xyz$ is fixed to the moving platform. In the base coordinate system of the fixed platform, the origin O1 is located at the midpoint of N2 and N3 on the fixed platform, with the *Z*-axis perpendicular to the fixed platform and oriented upwards as the positive direction. The *X*-axis is collinear with N2 and N3 and oriented towards N3 as the positive direction. The *Y*-axis is determined according to the right-hand rule. The origin O2 of the coordinate system fixed to the moving platform is located at the geometric center of the “匚”-shaped moving platform. In the initial state, the *x*-axis, *y*-axis, and *z*-axis are parallel to the *X*-axis, *Y*-axis, and *Z*-axis of the base coordinate system $O_{1}-XYZ$, as shown in Figure 6. Since the mechanism only exhibits three-dimensional translational motion characteristics, the axes in coordinate systems $O_{1}-XYZ$ and $O_{2}-xyz$ remain correspondingly parallel.

The centers of the first prismatic joints in the three branches are denoted as A1, A2, and A3, respectively. The first revolute joints in the three branches are C1, C2, and C3, with the axes of C1 and C2 being parallel and intersecting perpendicularly with the axis of C3 at points M1 and M2, respectively. M3 in Figure 6 is the midpoint of M1 and M2. The second revolute joints in the three branches are B1, B2, and B3. Therefore, the coordinates of O1 can be represented as $O_{1}=(0\;0\;0)$; the coordinates of O2 can be represented as $O_{2}=(P_{x}\;P_{y}\;P_{z})$, from which $\vec{O_{1}O_{2}}=(P_{x}\;P_{y}\;P_{z})$ can be inferred.

To determine the geometric parameters of the “匚”-shaped moving platform, the lengths of the “two horizontal bars” are both set to 2b, and the length of the “vertical bar” is 2a. In the moving coordinate system $O_{2}-xyz$, the ends B1, B2, and B3 of the three branches are located at the midpoints of the three sides of the “匚”-shaped moving platform. The coordinates $O_{2}-xyz$ of points B1, B2, and B3 in the moving coordinate system are obtained as follows:

(2) $$\left\{\begin{array}{l} O_{2}B_{1}=(\begin{array}{lll} -a & 0 & 0 \end{array})\\ O_{2}B_{2}=(\begin{array}{lll} a & 0 & 0 \end{array})\\ O_{2}B_{3}=(\begin{array}{lll} 0 & -b & 0 \end{array}) \end{array}\right.$$

Hence, we can obtain the vector.

(3) $$\left\{\begin{array}{l} \vec{O_{2}B_{1}}=(\begin{array}{lll} -a & 0 & 0 \end{array})\\ \vec{O_{2}B_{2}}=(\begin{array}{lll} a & 0 & 0 \end{array})\\ \vec{O_{2}B_{3}}=(\begin{array}{lll} 0 & -b & 0 \end{array}) \end{array}\right.$$

According to the rules of vector addition, we can obtain:

(4) $$\left\{\begin{array}{l} \vec{O_{1}B_{1}}=\vec{O_{1}O_{2}}+\vec{O_{2}B_{1}}=(\begin{array}{lll} P_{x}-a & P_{y} & P_{z} \end{array})\\ \vec{O_{1}B_{2}}=\vec{O_{1}O_{2}}+\vec{O_{2}B_{2}}=(\begin{array}{lll} P_{x}+a & P_{y} & P_{z} \end{array})\\ \vec{O_{1}B_{3}}=\vec{O_{1}O_{2}}+\vec{O_{2}B_{3}}=(\begin{array}{lll} P_{x} & P_{y}-b & P_{z} \end{array}) \end{array}\right.$$

To determine the geometric parameters of the fixed platform shaped like a “匚” (a frame with one vertical and two horizontal bars), we take the length of the vertical bar as L, and the lengths of the two horizontal bars are not specified, so we arbitrarily choose $A_{1}C_{1}=A_{2}C_{2}=A_{3}C_{3}=c$. Let the distance between A1 and N2 be d1, the distance between A2 and N3 be d2, and the distance between O1 and A3 be d3. Based on the aforementioned geometric relationships, the following vector information can be obtained:

(5) $$\left\{\begin{array}{l} \vec{O_{1}C_{1}}=(\begin{array}{lll} -L/2+c & d_{1} & 0 \end{array})\\ \vec{O_{1}C_{2}}=(\begin{array}{lll} L/2-c & d_{2} & 0 \end{array})\\ \vec{O_{1}C_{3}}=(\begin{array}{lll} d_{3} & c & 0 \end{array}) \end{array}\right.$$

(6) $$\left\{\begin{array}{l} \vec{O_{1}M_{1}}=(\begin{array}{lll} -L/2+c & c & 0 \end{array})\\ \vec{O_{1}M_{2}}=(\begin{array}{lll} L/2-c & c & 0 \end{array})\\ \vec{O_{1}M_{3}}=(\begin{array}{lll} 0 & c & 0 \end{array}) \end{array}\right.$$

Similarly, through vector addition, the following vector parameters can be obtained:

(7) $$\left\{\begin{array}{l} \vec{M_{1}B_{1}}=\vec{O_{1}B_{1}}-\vec{O_{1}M_{1}}=(\begin{array}{ccc} P_{x}+L/2-a-c & P_{y}-c & P_{z} \end{array})\\ \vec{M_{2}B_{2}}=\vec{O_{1}B_{2}}-\vec{O_{1}M_{2}}=(\begin{array}{ccc} P_{x}-L/2+a+c & P_{y}-c & P_{z} \end{array})\\ \vec{M_{3}B_{3}}=\vec{O_{1}B_{3}}-\vec{O_{1}M_{3}}=(\begin{array}{ccc} P_{x} & P_{y}-b-c & P_{z} \end{array}) \end{array}\right.$$

(8) $$\left\{\begin{array}{l} \vec{M_{1}C_{1}}=\vec{O_{1}C_{1}}-\vec{O_{1}M_{1}}=(\begin{array}{ccc} 0 & d_{1}-c & 0 \end{array})\\ \vec{M_{2}C_{2}}=\vec{O_{1}C_{2}}-\vec{O_{1}M_{2}}=(\begin{array}{ccc} 0 & d_{2}-c & 0 \end{array})\\ \vec{M_{3}C_{3}}=\vec{O_{1}C_{3}}-\vec{O_{1}M_{3}}=(\begin{array}{ccc} d_{3} & 0 & 0 \end{array}) \end{array}\right.$$

**Figure 7:**
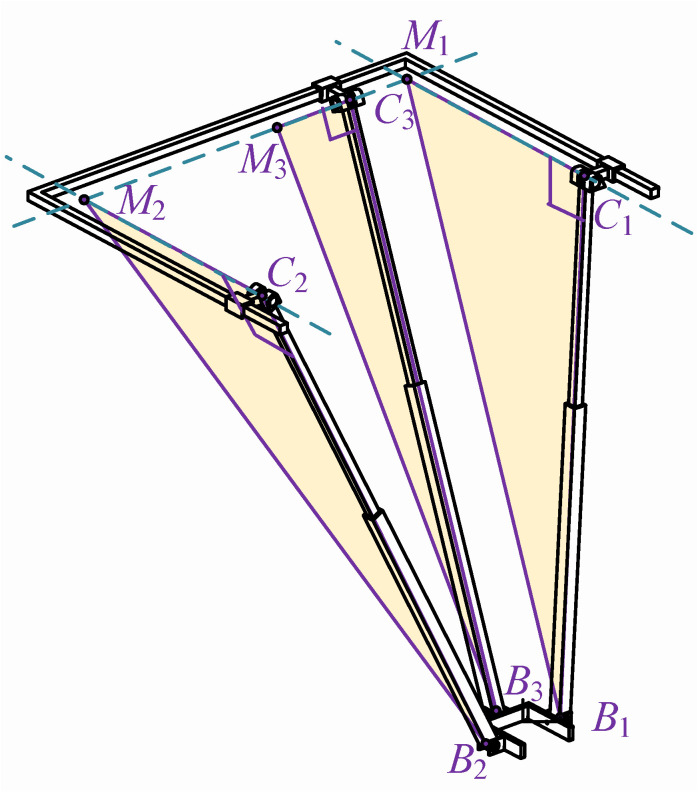
Geometric constraint diagram of the rehabilitation actuator.

When the mechanism is not in its initial configuration, as shown in Figure 7, the figure contains three right-angled triangles—C1M1B1, C2M2B2, and C3M3B3—which represent the fundamental geometric constraints of the mechanism. These constraints ensure precise positioning and accurate motion trajectories during the movement of the mechanism. The geometric relationships of each triangle are defined by constraint equations (such as Equation (9)), ensuring coordinated motion among different components. Even in certain special cases (e.g., when points C3 and M3 coincide or when points C1 and C2 coincide with M1 and M2), these constraints remain valid, thereby guaranteeing the stability and operability of the mechanism under various working conditions. $C_{1}M_{1}⊥C_{1}B_{1}$, $C_{2}M_{2}⊥C_{2}B_{2}$, $C_{3}M_{3}⊥C_{3}B_{3}$. Therefore, the triangles labeled $C_{1}M_{1}B_{1}$, $C_{2}M_{2}B_{2}$, and $C_{3}M_{3}B_{3}$ in the figure are all right triangles.

Considering the geometric constraint conditions of the three right-angle triangle structures C1, M1, and B1; C2, M2, and B2; and C3, M3, and B3, the following constraint equations can be derived:

(9) $$\left\{\begin{array}{l} {\vec{M_{1}C_{1}}}^{2}+{\vec{C_{1}B_{1}}}^{2}={\vec{M_{1}B_{1}}}^{2}\\ {\vec{M_{2}C_{2}}}^{2}+{\vec{C_{2}B_{2}}}^{2}={\vec{M_{2}B_{2}}}^{2}\\ {\vec{M_{3}C_{3}}}^{2}+{\vec{C_{3}B_{3}}}^{2}={\vec{M_{3}B_{3}}}^{2} \end{array}\right.$$

When the points C3 and M3 of the mechanism coincide, or when the points C1 and C2 coincide with the points M1 and M2, the mechanism does not form the right-angle triangles shown in Figure 7. However, it still satisfies the constraint equations in Equation (9). Therefore, the constraint equations are applicable to any configuration of the mechanism. By solving the system of equations, the following results can be obtained:

(10) $$\left\{\begin{array}{l} d_{1}=P_{y}\\ d_{2}=P_{y}\\ d_{3}=P_{x} \end{array}\right.$$

From the results of Equation (10), it can be inferred that the motions of the first prismatic joints A1 and A2 of the two branches, namely A1, C1, D1, and B1, on the one hand, and A2, C2, D2, and B2 (see Figure 6), which are symmetric in terms of a single plane, are coupled, and the components of the displacements of the two prismatic joints are the same. The component value is the same as the Y-direction component of the position information of the moving platform’s coordinate origin. The component of the first prismatic joint in the third branch is the same as the X-direction component of the position information center of the moving platform’s coordinate origin. Therefore, the first prismatic joints in the three branches are not suitable for being designed as the driving elements of the mechanism, and the selection must be made among the other kinematic pairs. Choosing the first revolute joints C1, C2, and C3, as well as the second revolute joints B1, B2, and B3, as the driving elements of the mechanism would make the mechanism bulky and increase its cost due to the design of the reducers. Therefore, the second prismatic joints D1, D2, and D3 in the three branches are selected as the driving parts of the overall mechanism, and using electric actuators to replace the prismatic joints can achieve good results.

To obtain the inverse kinematics of the mechanism, given the pose information of the moving platform’s coordinate system as $O_{2}=(P_{x}\;P_{y}\;P_{z})$ $O_{2}=(\begin{array}{ccc} P_{x} & P_{y} & P_{z} \end{array})$, we need to find the input values for the driving joints D1, D2, and D3. By selecting the second prismatic joints of each branch as the driving joints, which correspond to the lengths of C1, B1, C2, and B2 and of C3 and B3, and based on the previous calculations, the lengths of the three push rods can be obtained as follows:

(11) $$\left\{\begin{array}{l} L_{1}=\sqrt{{\vec{C_{1}B_{1}}}^{2}}=\sqrt{{\left(P_{x}+L/2-a-c\right)}^{2}+P_{z}^{2}+{\left(P_{y}-d_{1}\right)}^{2}}\\ L_{2}=\sqrt{{\vec{C_{2}B_{2}}}^{2}}=\sqrt{{\left(P_{x}-L/2+a+c\right)}^{2}+P_{z}^{2}+{\left(P_{y}-d_{2}\right)}^{2}}\\ L_{3}=\sqrt{{\vec{C_{3}B_{3}}}^{2}}=\sqrt{{\left(b-P_{y}+c\right)}^{2}+P_{z}^{2}+{\left(P_{x}-d_{3}\right)}^{2}} \end{array}\right.$$

Using SolidWorks2017 software, a 3D model of the rehabilitation mechanism was created. Given the three positional parameters Px, Py, and Pz of the moving platform, the lengths L1, L2, and L3 of the three driving push rods in the 3D model can be measured using the software. Based on the inverse kinematics expression of the mechanism calculated from Equation (11), the lengths of the three push rods were determined for the same pose parameters of the moving platform. These calculated lengths were then compared with the measured results from the model, and the results are presented in Table 7.

Through verification at three positions, it is evident that the measured values in the model perfectly match the theoretical values calculated from the inverse kinematics, confirming the correctness of the derived inverse kinematics for the mechanism.

To further verify the continuity of the inverse kinematics solution, Adams software can be utilized. By specifying a certain trajectory for the moving platform of the mechanism, the changes in the displacement components of the three driving joints in the Cartesian coordinate system can be measured. Then, the theoretical values can be calculated using the inverse kinematics formula, and the two sets of data can be compared.

After importing the software model into Adams and adding the corresponding constraints, the initial position of the mechanism needs to be determined. Here, the coordinate origin of the moving platform is set to $O_{2}=(0\;485-1225)$. A continuous motion is assigned to the moving platform, causing it to move upward along the axis at a constant speed of 300 mm over 5 s. Since Adams software can only measure the changes in the components of the three driving joints along the X, Y, and Z axes during this process, the length changes of the three driving branches need to be calculated using the inverse kinematics formula, which requires projection onto the X, Y, and Z axes. Some points during this motion are selected, with one point taken every 0.5 s in chronological order, resulting in 10 discrete points for verification. Then, the two sets of data are compared.

**Figure 8:**
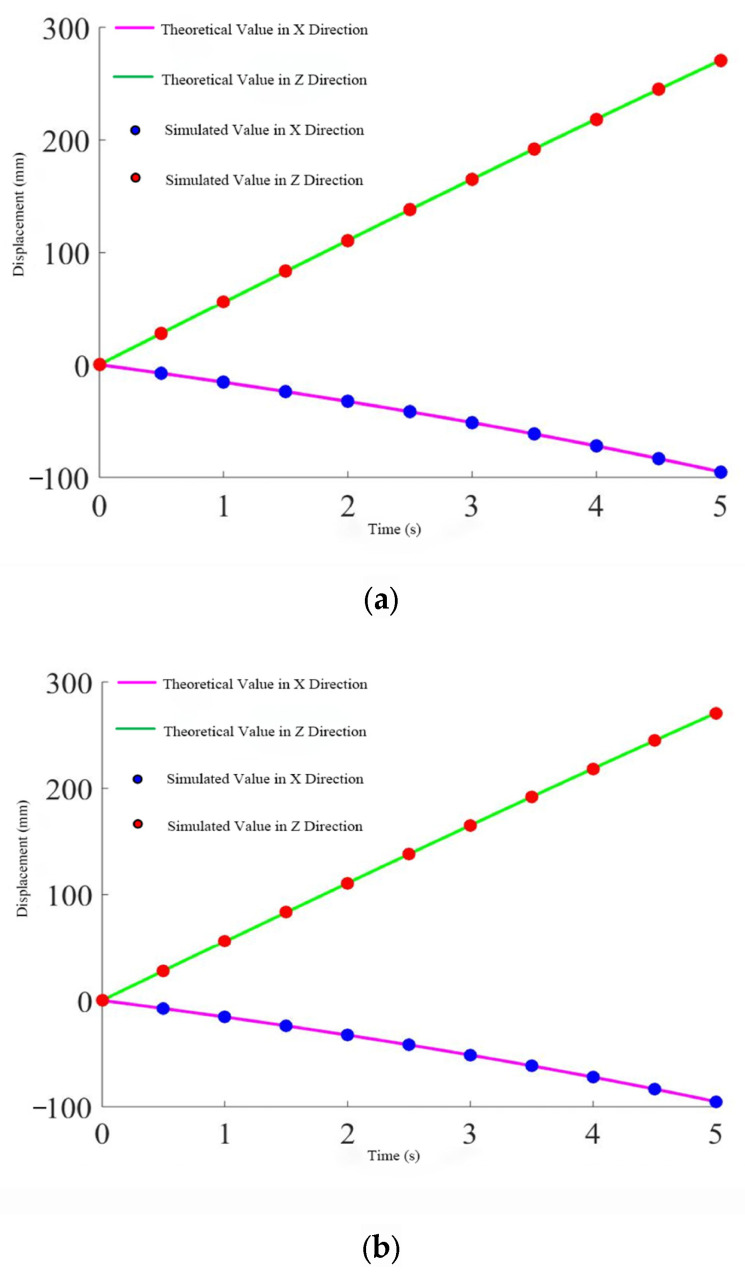

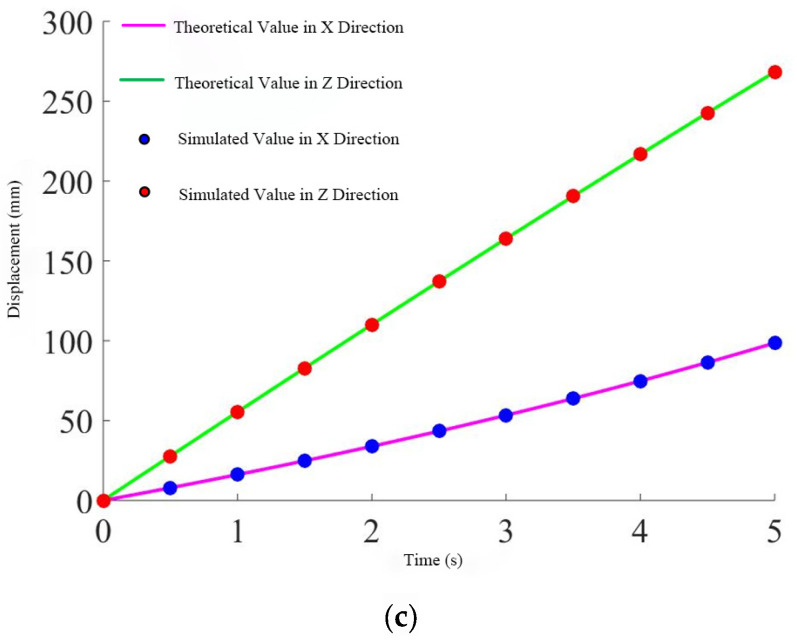
Displacement change of the push rod. (**a**) Rod one. (**b**) Rod two. (**c**) Rod three.

Based on the geometric relationship shown in Figure 6, it can be analyzed that the driving joints of branch one (A1, C1, D1, B1) and branch two (A2, C2, D2, B2) are always parallel to the plane XO1Z, while the driving joint of branch three is always parallel to the plane YO1Z. Therefore, the driving joints of branch one and branch two have no component in the *Y*-axis direction, and the driving joint of branch three has no component in the *X*-axis direction. The specific comparison results are shown in Figure 8.

From the three figures, it can be seen that the simulation values perfectly match the theoretical calculations, further proving the correctness and continuity of the derived inverse solution.

## 6. Conclusions

This study proposes an innovative design scheme for a multidimensional gait lower limb rehabilitation trainer based on a Bayesian network and a parallel mechanism, tailored to the rehabilitation needs of patients with lower limb motor dysfunction. By constructing a Bayesian network model of high-weight influencing factors and combining it with the kinematic decoupling characteristics of the parallel mechanism, a rehabilitation trainer with a multidimensional gait compensation function has been developed. This device not only effectively improves patients’ gait, enhances strength, and restores flexibility but also verifies the scientificity and effectiveness of its design through three-dimensional digital prototypes and human factors simulation. The rehabilitation needs of patients are thoroughly analyzed, and the Bayesian network model is used to assess the uncertainties in the rehabilitation process quantitatively. By defining functional impact weights and rehabilitation priorities, we provide a scientific basis for formulating personalized rehabilitation plans. Furthermore, through kinematic analysis and trajectory planning of the parallel mechanism, the smoothness and stability of the rehabilitation trainer’s movement are ensured, enhancing the patient’s rehabilitation experience. The research results indicate that the rehabilitation trainer designed in this study can drive the human lower limbs to perform various modes of rehabilitation exercises without being restricted to a plane, achieving comprehensive lower limb rehabilitation training. At the same time, the device also exhibits good applicability and convenience, adapting to patients of different body types and significantly improving the practicality of the rehabilitation robot.

## 7. Discussion

This study proposes a Bayesian network-based parallel-structure multidimensional gait lower limb rehabilitation trainer which exhibits significant innovation compared to traditional serial mechanisms and simple mechanical structures. Conventional rehabilitation devices typically follow fixed motion trajectories, limiting their ability to accommodate personalized rehabilitation needs. In contrast, this study employs a parallel mechanism that enables spatial three-degree-of-freedom translational motion, enhancing the multidimensionality of lower limb movement. Additionally, by integrating a Bayesian network, the system achieves real-time intelligent adjustments to training programs, significantly improving adaptability. Notably, it demonstrates superior flexibility and adaptability in addressing the J-curve motion pattern of the knee joint.

However, the current device remains in its early development stage and faces several technical and experimental challenges. First, although the parallel mechanism enhances motion flexibility, the risk of joint overload necessitates further optimization of precision and flexibility. This imposes stringent requirements on the durability of materials, transmission systems, and actuation mechanisms. As a result, repeated adjustments are necessary during experimental trials to ensure the system’s stability and functional reliability. Second, user experiments involving hemiplegic and stroke patients must strictly adhere to ethical review protocols and safety assessments to ensure patient well-being. This introduces additional requirements for both experimental design and device improvement.

Nevertheless, preliminary validation of the device’s feasibility has been achieved through parallel lower limb mechanism modeling and simulation analysis. Future research will focus on comparative experiments, particularly benchmarking against traditional rehabilitation methods and other advanced technologies. Key performance indicators, such as rehabilitation efficiency and patient adaptability, will be analyzed to further assess the system’s practical effectiveness. Furthermore, as the device undergoes continued optimization and accumulates more clinical trial data, its reliability and efficacy are expected to be substantiated, promoting its widespread application in lower limb rehabilitation and laying the foundation for industrialization.

## Figures and Tables

**Table 1:** Prior probability distribution of the hip joint observation node.

B11 Flexion (Hip)	B12 Extension (Hip)	B21 Abduction (Hip)	B22 Adduction (Hip)	B31 External Rotation (Hip)	B32 Internal Rotation (Hip)	P1 Rehabilitation Goal (Hip)		
						Gait Improvement	Strength Enhancement	Flexibility Restoration
State0	State0	State0	State0	State0	State0	0.6	0.25	0.15
					State1	0.18	0.35	0.47
				State1	State0	0.56	0.23	0.21
					State1	0.2	0.57	0.23
			State1	State0	State0	0.72	0.14	0.14
					State1	0.22	0.46	0.32
				State1	State0	0.57	0.25	0.18
					State1	0.22	0.62	0.16
		State1	State0	State0	State0	0.6	0.25	0.15
					State1	0.14	0.59	0.27
				State1	State0	0.48	0.39	0.13
					State1	0.25	0.45	0.3
			State1	State0	State0	0.64	0.21	0.15
					State1	0.21	0.64	0.15
				State1	State0	0.37	0.32	0.31
					State1	0.15	0.75	0.1
	State1	State0	State0	State0	State0	0.82	0.12	0.06
					State1	0.27	0.65	0.08
				State1	State0	0.55	0.23	0.22
					State1	0.24	0.58	0.18
			State1	State0	State0	0.33	0.33	0.34
					State1	0.27	0.23	0.5
				State1	State0	0.31	0.34	0.35
					State1	0.48	0.5	0.02
		State1	State0	State0	State0	0.37	0.26	0.37
					State1	0.11	0.14	0.75
				State1	State0	0.41	0.27	0.32
					State1	0.05	0.5	0.45
			State1	State0	State0	0.27	0.36	0.37
					State1	0.05	0.82	0.13
				State1	State0	0.41	0.41	0.18
					State1	0.25	0.69	0.06
State1	State0	State0	State0	State0	State0	0.26	0.35	0.39
					State1	0.41	0.27	0.32
				State1	State0	0.19	0.53	0.28
					State1	0.4	0.4	0.2
			State1	State0	State0	0.44	0.28	0.28
					State1	0.3	0.43	0.27
				State1	State0	0.29	0.3	0.41
					State1	0.1	0.63	0.27
		State1	State0	State0	State0	0.49	0.1	0.41
					State1	0.25	0.45	0.3
				State1	State0	0.34	0.28	0.38
					State1	0.35	0.35	0.3
			State1	State0	State0	0.24	0.38	0.38
					State1	0.12	0.45	0.43
				State1	State0	0.3	0.68	0.02
					State1	0.2	0.25	0.55
	State1	State0	State0	State0	State0	0.29	0.36	0.35
					State1	0.41	0.39	0.2
				State1	State0	0.3	0.25	0.45
					State1	0.09	0.5	0.41
			State1	State0	State0	0.37	0.5	0.13
					State1	0.3	0.22	0.48
				State1	State0	0.72	0.14	0.14
					State1	0.18	0.27	0.55
		State1	State0	State0	State0	0.27	0.37	0.36
					State1	0.06	0.81	0.13
				State1	State0	0.4	0.4	0.2
					State1	0.25	0.69	0.06
			State1	State0	State0	0.56	0.24	0.2
					State1	0.24	0.57	0.19
				State1	State0	0.64	0.21	0.15
					State1	0.48	0.5	0.02

**Table 2:** Prior probability distribution of the knee joint observation node.

B41 Flexion (Knee)	B51 External Rotation (Knee)	B52 Internal Rotation (Knee)	P2 Rehabilitation Goals (Knee)		
			Enhanced Coordination	Increased Strength	Improved Flexibility
State0	State0	State0	0.51	0.26	0.23
		State1	0.27	0.55	0.18
	State1	State0	0.47	0.35	0.18
		State1	0.25	0.57	0.18
State1	State0	State0	0.31	0.34	0.35
		State1	0.38	0.28	0.34
	State1	State0	0.41	0.33	0.26
		State1	0.29	0.37	0.34

**Table 3:** Prior probability distribution of the ankle joint observation node.

B61 Flexion (Ankle)	B62 Dorsiflexion (Ankle)	B71 Abduction (Ankle)	B72 Adduction (Ankle)	B81 External Rotation (Ankle)	B82 Internal Rotation (Ankle)	P3 Rehabilitation Goals (Ankle)		
						Improved Balance Ability	Enhanced Motor Ability	Increased Physical Fitness
State0	State0	State0	State0	State0	State0	0.17	0.46	0.37
					State1	0.53	0.27	0.2
				State1	State0	0.27	0.3	0.43
					State1	0.35	0.22	0.43
			State1	State0	State0	0.2	0.69	0.11
					State1	0.2	0.31	0.49
				State1	State0	0.22	0.38	0.4
					State1	0.58	0.3	0.12
		State1	State0	State0	State0	0.31	0.29	0.4
					State1	0.49	0.31	0.2
				State1	State0	0.2	0.4	0.4
					State1	0.14	0.46	0.4
			State1	State0	State0	0.29	0.15	0.56
					State1	0.11	0.69	0.2
				State1	State0	0.48	0.04	0.48
					State1	0.33	0.37	0.3
	State1	State0	State0	State0	State0	0.05	0.55	0.4
					State1	0.85	0.14	0.01
				State1	State0	0.1	0.3	0.6
					State1	0.3	0.35	0.35
			State1	State0	State0	0.09	0.51	0.4
					State1	0.71	0.19	0.1
				State1	State0	0.2	0.3	0.5
					State1	0.33	0.27	0.4
		State1	State0	State0	State0	0.66	0.24	0.1
					State1	0.2	0.3	0.5
				State1	State0	0.24	0.36	0.4
					State1	0.56	0.34	0.1
			State1	State0	State0	0.28	0.36	0.36
					State1	0.05	0.85	0.1
				State1	State0	0.44	0.1	0.46
					State1	0.31	0.38	0.31
State1	State0	State0	State0	State0	State0	0.4	0.45	0.15
					State1	0.3	0.2	0.5
				State1	State0	0.15	0.4	0.45
					State1	0.29	0.7	0.01
			State1	State0	State0	0.47	0.33	0.2
					State1	0.2	0.4	0.4
				State1	State0	0.16	0.44	0.4
					State1	0.64	0.26	0.1
		State1	State0	State0	State0	0.2	0.3	0.5
					State1	0.41	0.27	0.32
				State1	State0	0.09	0.73	0.18
					State1	0.5	0.48	0.02
			State1	State0	State0	0.2	0.32	0.48
					State1	0.1	0.73	0.17
				State1	State0	0.48	0.11	0.41
					State1	0.41	0.5	0.09
	State1	State0	State0	State0	State0	0.15	0.3	0.55
					State1	0.25	0.38	0.37
				State1	State0	0.55	0.32	0.13
					State1	0.11	0.49	0.4
			State1	State0	State0	0.2	0.3	0.5
					State1	0.08	0.52	0.4
				State1	State0	0.72	0.18	0.1
					State1	0.2	0.3	0.5
		State1	State0	State0	State0	0.4	0.3	0.3
					State1	0.19	0.43	0.38
				State1	State0	0.22	0.58	0.2
					State1	0.37	0.15	0.48
			State1	State0	State0	0.27	0.2	0.53
					State1	0.32	0.3	0.38
				State1	State0	0.32	0.2	0.48
					State1	0.41	0.25	0.34

**Table 4:** Posterior probability distribution of the hip, knee, and ankle joint observation nodes.

Rehabilitation Training	Rehabilitation Goals (Hip)			Rehabilitation Goals (Knee)			Rehabilitation Goals (Ankle)		
	Improved Gait	Strength Enhancement	Flexibility Restoration	Flexibility Restoration	Increased Strength	Improved Flexibility	Improved Balance Ability	Enhanced Motor Ability	Increased Physical Fitness
Minor Repair	0.410	0.350	0.240	0.416	0.350	0.234	0.278	0.384	0.337
Minor Repair	0.399	0.357	0.244	0.415	0.348	0.236	0.288	0.377	0.334
Major Repair	0.357	0.380	0.262	0.374	0.382	0.244	0.312	0.364	0.325

**Table 5:** Fatigue protection design requirements for multidimensional gait lower limb rehabilitation trainers.

Serial Number	Description of Requirement	Importance (Average Value)
01	Effectively supports the lower limbs and reduces joint burden.	4.8
02	Able to alleviate muscle fatigue in the waist and back of the trainee.	4.5
03	The equipment is comfortable to wear and does not restrict normal breathing of the trainee.	4.0
04	Equipment design ensures comfort for prolonged use.	4.2
05	Adjustable sizing to accommodate the body shapes of different patients.	4.1
06	Material is breathable and skin-friendly, reducing skin irritation.	3.7
07	The device is lightweight, facilitating easy wearing and movement for patients.	3.9
08	Operation is simple and intuitive, making it easy for patients to use.	4.3
09	The equipment has sufficient stability to ensure training safety.	4.6
10	Good wearing stability to prevent slipping during training.	4.4

**Table 6:** Range of motion for the three major joints of the human lower limbs.

Joint Name	Movement Parameters	Physiological Range of Motion (ROM)	Normal Gait Range
Hip joint	Sagittal plane (flexion/extension)	0–140/0–15	0–40/0–5
	Coronal plane (abduction/adduction)	0–30/0–25	0–5/0–3
	Horizontal plane (external rotation/internal rotation)	0–90/070	0–7/0–3
Knee joint	Sagittal plane (flexion/extension)	0–140/0	0–67/0
	Coronal plane (abduction/adduction)	--	--
	Horizontal plane (external rotation/internal rotation)	0–45/0–30	0–8/0–8
Ankle joint	Sagittal plane (flexion/extension)	0–30/0–45	0–7/0–10
	Coronal plane (abduction/adduction)	0–15/0–20	0–3/0–3
	Horizontal plane (external rotation/internal rotation)	0–15/0–15	0–3/0–3

**Table 7:** Verification of the inverse kinematics position of the mechanism.

Verification Position (mm) Px, Py, Pz	SolidWorks Model (mm) L1, L2, L3	Theoretical Calculation (mm) L1, L2, L3
300, 400, −1000 200, 350, −900 −400, 450, −950	1187.26, 1000.80, 1028.39 1049.57, 910.82, 904.49 951.89, 1204.20, 950.05	1187.26, 1000.80, 1028.39 1049.57, 910.82, 904.49 951.89, 1204.20, 950.05

## Data Availability

The raw data supporting the conclusions of this article will be made available by the authors on request.

## References

[1] Khan N.A., Hussain S., Spratford W., Goecke R., Kotecha K., Jamwal P.K. (2025). Deep Learning-Driven Analysis of a Six-Bar Mechanism for Personalized Gait Rehabilitation. J. Comput. Inf. Sci. Eng..

[2] Kapsalyamov A., Brown N.A.T., Goecke R., Jamwal P.K., Hussain S. (2025). Velocity Control of a Stephenson III Six-Bar Linkage-Based Gait Rehabilitation Robot Using Deep Reinforcement Learning. Neural Comput. Appl..

[3] Shi D., Zhang W., Zhang W., Ding X. (2019). A Review on Lower Limb Rehabilitation Exoskeleton Robots. Chin. J. Mech. Eng..

[4] Zhang X., Yue Z., Wang J. (2017). Robotics in Lower-Limb Rehabilitation after Stroke. Behav. Neurol..

[5] Torun H.M., Swaminathan M., Kavungal Davis A., Bellaredj M.L.F. (2018). A Global Bayesian Optimization Algorithm and Its Application to Integrated System Design. IEEE Trans. VLSI Syst..

[6] Bensi M., Kiureghian A.D., Straub D. (2013). Efficient Bayesian Network Modeling of Systems. Reliab. Eng. Syst. Saf..

[7] Freedman H., Metzger J., Abolhassani N., Tudor A., Tomlinson B., Paul S. (2024). A Bayesian Approach to Constructing Probabilistic Models from Knowledge Graphs. Int. J. Semant. Comput..

[8] Ziegler Haselein B., Da Silva J.C., Hooey B.L. (2024). Multiple Machine Learning Modeling on near Mid-Air Collisions: An Approach towards Probabilistic Reasoning. Reliab. Eng. Syst. Saf..

[9] Yu C., Liu J., Nemati S., Yin G. (2023). Reinforcement Learning in Healthcare: A Survey. ACM Comput. Surv..

[10] Shen T., Zhang M., Wu T. (2024). Emotion-Driven Action Interaction Design in Small Quadruped Robots: Leveraging NXEIK Multilevel Network. J. Eng. Des..

[11] Hunte J.L., Neil M., Fenton N.E. (2024). A Hybrid Bayesian Network for Medical Device Risk Assessment and Management. Reliab. Eng. Syst. Saf..

[12] Wang B., Chen Y., Li Z. (2024). A Novel Bayesian Pay-As-You-Drive Insurance Model with Risk Prediction and Causal Mapping. Decis. Anal. J..

[13] Vieider F.M. (2024). Decisions Under Uncertainty as Bayesian Inference on Choice Options. Manag. Sci..

[14] Philip B., AlJassmi H. (2024). A Bayesian Decision Support System for Optimizing Pavement Management Programs. Heliyon.

[15] Mitake Y., Inagaki Y., Tsuji S., Shimomura Y. (2024). Identification of the Causal Relationship between Features and Barriers of Product–Service Systems Based on Bayesian Network Model. Procedia CIRP.

[16] Guo J., Ma K. (2024). Risk Analysis for Hazardous Chemical Vehicle-Bridge Transportation System: A Dynamic Bayesian Network Model Incorporating Vehicle Dynamics. Reliab. Eng. Syst. Saf..

[17] Li G., Liu Z., Zhang J., Han H., Shu Z. (2024). Bayesian Model Averaging by Combining Deep Learning Models to Improve Lake Water Level Prediction. Sci. Total Environ..

[18] Laborda J.D., Torrijos P., Puerta J.M., Gámez J.A. (2024). Parallel Structural Learning of Bayesian Networks: Iterative Divide and Conquer Algorithm Based on Structural Fusion. Knowl.-Based Syst..

[19] Xu W., Futrell R. (2024). A Hierarchical Bayesian Model for Syntactic Priming. arXiv.

[20] Gao S., Chen J., Chen X., Uchitel J., Tang C., Li C., Pan Y., Zhao H. (2024). Temporal Dynamics and Physical Priori Multimodal Network for Rehabilitation Physical Training Evaluation. IEEE J. Biomed. Health Inform..

[21] Sun J., Hu F., Gao K., Gao F., Ma C., Wang J. (2024). Research and Experiment on Active Training of Lower Limb Based on Five-Bar Mechanism of Man-Machine Integration System. Robotica.

[22] Jin W., Liu J., Zhang Q., Zhang X., Wang Q., Xu J., Fang H. (2024). Forward Dynamics Simulation of a Simplified Neuromuscular-Skeletal-Exoskeletal Model Based on the CMA-ES Optimization Algorithm: Framework and Case Studies. Multibody Syst. Dyn..

[23] Qazi A., Simsekler M.C.E., Al-Mhdawi M.K.S. (2024). Bayesian Network and Structural Equation Modeling of Dependencies between Country-Level Sustainability Risks and Logistics Performance. Ann. Oper. Res..

[24] Herlambang Cahya Pratama Y., Al Hafidz M., Lazuardy N., Naristi K. (2024). Application Of User Centered Design (Ucd) Method For Ui/Ux Design At Husqy Petshop. MSJ.

[25] Hasim W., Wibirama S., Nugroho H.A. Redesign of E-Participation Using User-Centered Design Approach for Improving User Experience. Proceedings of the 2019 International Conference on Information and Communications Technology (ICOIACT).

[26] Lowe B.D., Dempsey P.G., Jones E.M. (2019). Ergonomics Assessment Methods Used by Ergonomics Professionals. Appl. Ergon..

[27] Yang T., Gao X., Gao R., Dai F., Peng J. (2019). A Novel Activity Recognition System for Alternative Control Strategies of a Lower Limb Rehabilitation Robot. Appl. Sci..

[28] Hernández-Ramírez R., Ayanoğlu H., Duarte E. (2019). On the Origins and Basic Aspects of User-Centered Design and User Experience. Emotional Design in Human-Robot Interaction.

[29] Maguire M., Soares M.M., Rosenzweig E., Marcus A. (2021). A Study of Student Creative Thinking in User-Centred Design. Design, User Experience, and Usability: UX Research and Design.

[30] Graham A.K., Wildes J.E., Reddy M., Munson S.A., Barr Taylor C., Mohr D.C. (2019). User-centered Design for Technology-enabled Services for Eating Disorders. Intl. J. Eat. Disord..

[31] Carmo A.A., Kleiner A.F.R., Costa P.H.L.D., Barros R.M.L. (2012). Three-Dimensional Kinematic Analysis of Upper and Lower Limb Motion during Gait of Post-Stroke Patients. Braz. J. Med. Biol. Res..

